# Working Memory Training and CBT Reduces Anxiety Symptoms and Attentional Biases to Threat: A Preliminary Study

**DOI:** 10.3389/fpsyg.2016.00047

**Published:** 2016-02-02

**Authors:** Julie A. Hadwin, Helen J. Richards

**Affiliations:** Developmental Brain-Behaviour Laboratory, School of Psychology, University of SouthamptonSouthampton, UK

**Keywords:** anxiety, working memory, attentional control, intervention, attentional bias to threat, randomized control trial

## Abstract

Research indicates that cognitive processes linked to the detection of threat stimuli are associated with poor attentional control, placing children and adolescents at increased risk for the development of anxious affect. The current study aimed to provide preliminary data to assess whether an intervention designed to improve attentional control (via working memory; WM) would lead to better performance in tests of WM and would be associated with positive changes in symptoms of trait and test anxiety, increased inhibitory control and reduced attention to threat. Forty adolescents aged 11–14 years who reported elevated anxiety and low attentional control were randomly allocated to a WM training or an active cognitive behavioural therapy (CBT) control group. Post intervention, WM training was associated with greater improvements (versus. CBT) in trained WM tasks. Both groups, however, reported fewer anxiety symptoms, demonstrated increased inhibitory control and a reduction in attentional biases to threat post intervention and these results were maintained at follow up. The study provides indicative evidence which suggests that WM training has similar benefits to a more traditional CBT intervention on reduced anxiety and attentional biases for threat. Future research should aim to replicate the findings in a large sample size and explore the broader impact of training on day-to-day functioning. In addition, further research is needed to identify which participants benefit most from different interventions (using baseline characteristics) on treatment compliance and outcome.

## Introduction

Research suggests that clinical levels of anxiety are experienced by 2–15% of children and adolescents (Rapee et al., 2009). Anxiety follows a chronic pathway through development and is associated with several negative outcomes including lowered attendance at school (Richards and Hadwin, 2011; Wood et al., 2012), educational underachievement (Owens et al., 2008), poor peer relationships (Asendorpf et al., 2008) and increased risk for further mental and physical health difficulties (Roza et al., 2003). Researchers recognize that increased recruitment of attentional processes linked to regions of the prefrontal cortex (PFC) are important in the regulation of negative affect (e.g., Banks et al., 2007; Hare et al., 2008; review by Graham and Milad, 2013). Theoretical frameworks of anxiety have increasingly focused on poor attentional control as one cognitive mechanism involved in the onset and maintenance of anxious affect (Eysenck and Calvo, 1992; Eysenck et al., 2007).

Cognitive theories aim to understand the nature and impact of anxiety-related impairments in attentional control on performance in cognitive tasks and on attention in daily life. Attentional Control Theory (Eysenck et al., 2007; Eysenck and Derakshan, 2011) for example, proposes that anxiety impacts core cognitive processes linked to inhibitory control (to resist interference from non-relevant distractors), set shifting (to move attention between relevant information or location) and updating information in WM (to remember and revise material for further processing). It suggests that the negative association between anxiety and cognition is most evident when attentional resources are directed toward external or internal threatening stimuli. Related theories similarly propose that elevated anxiety is associated with increased attention towards threat stimuli (e.g., Bar-Haim et al., 2007). Researchers have also argued that the allocation of attention to threat in anxiety leads to subsequent avoidance that works to help individuals regulate feelings of negative affect in the short term (Mogg and Bradley, 1998).

A substantial body of research has shown that children and adolescents with elevated anxiety rapidly focus attention on threat stimuli and demonstrate difficulties inhibiting task-irrelevant threat (Hare et al., 2008; Hadwin et al., 2009; Nightingale et al., 2010; Waters et al., 2012; review by Dudeney et al., 2015). Further research has found evidence of threat avoidance in childhood (Stirling et al., 2006) and adult anxiety (Horley et al., 2004). Attention processes associated with anxious affect have also been shown to predict anxiety over time in development. For example, poor attentional control at 6 years of age was associated with high stable and increasing anxiety trajectories across middle to late childhood (Duchesne et al., 2010). In addition, threat biases to angry (versus happy faces; as indicated in enhanced N170 amplitudes) were found to predict increased anxiety over time in children aged 5–7 years (O’Toole et al., 2013). Further studies have found that good attentional control moderates threat biases in anxiety. Susa et al. (2012), for example, found that a positive association between anxiety and attentional bias to threat was only evident in 9–14-year-old participants who reported low (versus high) levels of attentional control (see also Lonigan and Vasey, 2009 for similar results).

Attention bias modification (ABM) techniques were developed to reduce attentional biases to threat in anxiety using experimental paradigms that direct attention away from threatening stimuli or toward positive stimuli and where the overall aim is to reduce symptoms of anxious affect. Recent studies have found that ABM leads to reductions in attentional biases for threat and anxiety symptoms in children and adolescents (e.g., Rozenman et al., 2011; Eldar et al., 2012). For example, Eldar et al. (2012) conducted a randomised controlled trial (RCT) where participants aged 8–14 years completed four sessions of ABM (versus a placebo condition) over a 4 week period. The results showed reductions in attentional threat bias and clinician reported anxiety across the intervention period in the ABM (versus the placebo) group. Although significant reductions in parent and child report anxiety occurred across both groups. While a recent review outlined that further research is needed to understand the effectiveness of ABM in the reduction of anxiety (Lowther and Newman, 2014), studies have provided preliminary support for the development of interventions in children and adolescents to target anxiety symptoms via reduced attention to threat.

Further interventions have aimed to increase attentional control to reduce symptoms of psychopathology in development. These have largely focused on increasing WM capacity to impact inattention symptoms in children and adolescents diagnosed with attention deficit hyperactivity disorder (e.g., Beck et al., 2010; see review by Rabipour and Raz, 2012). WM is defined as a limited capacity system made up of the phonological loop, the visuospatial sketchpad (processing verbal versus visual and spatial information respectively), the central executive and the episodic buffer (suggested to act as an interface between current cognitive processing with information stored in long term memory; Baddeley, 2003). The central executive component of WM is proposed to form part of prefrontal processes that underpin attentional control (Baddeley, 2003; Kane and McVay, 2012). WM training has been linked to increased activation in prefrontal and parietal brain regions when completing WM tasks (Klingberg et al., 2002; Olesen et al., 2003; review by Bunge and Wright, 2007).

A recent review of training studies highlighted their utility in improving WM as well as attentional control more broadly (Jaeggi and Buschkuehl, 2014). Consistently, a recent meta-analysis reviewed 622 studies using one WM training programme (CogMed) and found evidence of moderate benefits (compared to passive control groups post intervention) and small to moderate benefits (between groups at follow-up) in WM capacity and inattention in daily life as reported by parents and teachers (Spencer-Smith and Klingberg, 2015^1^). Similarly, a meta-analysis of WM and executive function training more broadly (versus passive control groups) were reported to have beneficial effects in older adults on training tasks and wider executive attention (Karbach and Verhaeghen, 2014). Despite broadly positive outcomes for WM training, other reviews have highlighted some challenges within this literature linked to the longevity of effects and transfer to novel WM tasks or intelligence more broadly (Melby-Lervåg and Hulme, 2013).

Few studies have examined the impact of WM training on symptoms of negative affect. Owens et al. (2013) showed that WM training in adults who reported elevated symptoms of depression was associated with increased WM capacity (compared to a low level active control group). This improvement was reflected in greater event related potential (ERP) amplitudes as measured in contralateral delay activity following the intervention and where this component occurs around 300 ms after stimulus onset and is argued to reflect increased retention of remembered items in visuospatial WM (see Ikkai et al., 2010). Consistent with this finding, an intervention study in 12- to 13-year-olds who were recognized to have social, emotional and behavioral difficulties demonstrated that a WM intervention (compared with a passive control group) increased performance in WM tasks and improved attentional control more broadly (as measured in a behavioral inhibition task). In addition, young people reported fewer symptoms of test anxiety following the intervention (Roughan and Hadwin, 2011). The possibility that a WM intervention can lower negative affect has significant implications for the development of translational research that increases attentional control to enable regulation of emotion and to allow individuals to meet goals in day-to-day life (see Bishop, 2007).

The current study aimed to provide preliminary data to test the proposition that interventions that work to increase attentional control (via improved WM) will have a positive effect on anxiety symptoms and attentional processes associated with negative affect (poor attentional control and attentional capture or avoidance of threat stimuli; Legerstee et al., 2010; Waters et al., 2012). Specifically, it assessed the impact of a WM intervention in adolescents who reported elevated anxiety symptoms and low attentional control. Previous work has reliably established that WM interventions show positive outcomes compared to passive control groups (review by Spencer-Smith and Klingberg, 2015). In addition, several researchers have highlighted that wait-list groups can overestimate treatment effects (e.g., Cunningham et al., 2013). Therefore, the current study compared a WM intervention to an active control group based on cognitive behavioural therapy (CBT); a widely accepted treatment of choice for anxiety (review by Cowart and Ollendick, 2010). Following previous research we anticipated that the CBT intervention would reduce anxiety symptoms in young people. We expected that the WM intervention should increase performance in WM tasks and that it would have a broader positive impact on key measures of attention (i.e., inhibitory control and attentional bias to threat), as well as feelings of anxious affect.

## Materials and Methods

### Participants

Participants were 11–14 year olds from four secondary schools in the UK. One thousand, five hundred and sixty young people were invited to complete screening questionnaires and 640 individuals agreed to participate with parental consent. The only exclusion criterion was the documented presence of special educational needs, leading to the exclusion of 14 young people. Following exclusions and based on the screening inclusion criteria (see below), we identified and approached 146 young people who were eligible to take part and 40 individuals provided written assent and written parental consent to participate. Participants (mean age = 13 years, 0 months; 10 males; *N* = 36 White, *N* = 1 Asian and *N* = 3 Mixed Race.) were randomized to receive one of the two interventions. Appendix A in Supplementary Material outlines the flow of participants through each phase of the study.

### Measures

#### Screening Measures

Participants completed two self-report questionnaires to assess anxiety and attentional control. Anxiety was measured using the 6-item generalized anxiety subscale from the Spence Children’s Anxiety Scale (SCAS; Spence, 1998), with possible scores ranging from 0 to 18. Internal consistency in the screened sample was good (α = 0.84). We screened attentional control using a 9-item questionnaire with possible scores ranging from 9 to 45; this measure included the seven items from the attention subscale on the Early Adolescent Temperament Questionnaire Revised (Ellis and Rothbart, 2001) and two additional items used to assess WM ability in a school or homework setting. The internal consistency of the scale in the screened sample was acceptable (α = 0.73).

Individuals eligible to take part in the interventions scored above average (T-score > 50) on the anxiety questionnaire based on age and gender appropriate norms (scores of 6 or more for males and 7 or more for females) and scored at or below the median for the screened sample (Median = 31, *n* = 640) on the attentional control questionnaire. In the final sample, 27 participants scored in the ‘elevated anxiety’ range on the SCAS (T-score > 60, corresponding to scores of 9 or more) and 13 participants scored above average but below the elevated anxiety level (T-score > 50). The mean anxiety score in the screened sample (*n* = 640) was 5.79 (*SD* = 3.92, range = 0–18) and in the intervention group the mean was 11.15 (*SD* = 3.52, range = 6–17). Considering attentional control, respective means in the screened and the intervention samples were 5.32 (*SD* = 5.32, range = 15–44) and 26.50 (*SD* = 26.50, range = 20–31).

#### Outcome Measures

Outcome measures were completed at three time points: prior to the intervention (Time 1 – T1); within 3 weeks after the intervention (Time 2 – T2); between 3 and 4 months after the intervention (Time 3 – T3)^2^ .

#### Working Memory

We measured near WM ability (tasks that were similar to taught tasks within the intervention) and distant WM ability (tasks that were not similar to taught tasks). Near tasks included a percentage correct composite score from the backward digit recall subtest and a modified (backward) version of the block recall subtest from the Working Memory Test Battery for Children (Pickering and Gathercole, 2001) to assess verbal and spatial WM, respectively. Participants heard a list of digits or saw a sequence of blocks of increasing length and were asked to repeat them in the reverse order.

Distant WM was assessed using the verbal and spatial versions of the computerized 2-n-back task (Shackman et al., 2006). In each task, an array of 34 letters was presented continuously for an entire block of trials. Each trial began with the appearance of a small square highlighting a subsection of the letters for 500 ms, followed by a 3500 ms interval in which the letter array was presented without the small square, followed immediately by the next trial. In the spatial task, participants pressed a button on each trial to indicate whether the location of the square was the same (matched trials) or different (mismatched trials) to the location of the square presented two trials earlier. In the verbal version, they indicated whether the type of letters inside the square was the same or different to the square presented two trials earlier. Each task included 18 matched trials and 54 mismatched trials. We calculated a composite score across tasks based on the percentage of accurate responses in the matched and mismatched trials.

#### Anxiety

The total score from the Revised Children’s Manifest Anxiety Scale (RCMAS 2nd Edition; Reynolds and Richmond, 2008) was used to assess anxiety. The scale is made up of 40 items assessing physiological anxiety, worry and social anxiety. Participants answer each item with a yes/no response, generating possible scores from 0 to 40. The internal consistency for the total scale in the current sample was excellent (α = 0.90, *n* = 40 at T1).

We used the total score from the Child Test Anxiety Scales to measure test anxiety (Wren and Benson, 2004). This scale includes 30 items to measure worry, physiological change and behaviors associated with taking tests. Participants are asked to indicate for each item (e.g., “I think most of my answers are wrong”) whether they “almost never” (1), “some of the time” (2), “most of the time” (3) “to almost always” (4) show that behavior. The total score ranges from 30 to 120.

#### Inhibitory Control

Participants completed a computerized Stroop paradigm (Stroop, 1935) with 108 experimental trials. A trial consisted of a fixation cross for 500 ms, followed by a single word (BLUE, YELLOW, RED, or GREEN) or a horizontal string of Xs that disappeared upon response, followed by a blank screen for 1000 ms. The stimuli were typed in blue, yellow, red, or green font and participants pressed a button as quickly as possible to indicate the color of the font. There were three trial conditions presented with equal frequency and in a random order: (1) Congruent trials in which the word content and font color were matched; (2) Incongruent trials in which the word content and font color were mismatched; (3) Neutral trials in which participants were presented with a string of Xs in colored font. The dependent variable was an interference score in which mean RTs in the congruent condition were subtracted from mean RTs in the incongruent condition; positive scores indicate interference from incongruent information.

#### Attentional Bias to Threat

Participants completed a computerized dot probe task (MacLeod et al., 1986) consisting of 72 experimental trials. A trial started with a fixation cross for 500 ms, followed by a pair of faces presented for 500 ms, followed by a probe stimulus (two small dots) in the location of one of the previous faces until response, followed by a blank screen for 1000 ms. Participants indicated the orientation of the small dots (horizontal or vertical) as quickly as possible with a button press. The task included angry or neutral expressions portrayed by four models (two male, two female) from the NimStim set of facial expressions (Tottenham et al., 2009). The pair of faces in each trial was either angry-neutral (48 trials) or neutral-neutral (24 trials). There were three trial conditions that occurred with equal frequency and in a random order, where the probe replaced: (1) the angry face in angry-neutral pairs (congruent trials); (2) the neutral face in angry-neutral pairs (incongruent trials); (3) either of the faces in the neutral–neutral pairs (neutral trials). The dependent variable was an attentional bias score, calculated by subtracting the mean RT in the congruent condition from the mean RT in the incongruent condition; a positive score indicates a bias toward threat and a negative score indicates a bias away from threat. Scores that tend toward 0 indicate less interference from facial stimuli to meet task goals.

#### IQ

An estimate of full scale IQ was generated at T1 only using the matrix reasoning and vocabulary subtests from the Wechsler Abbreviated Scale of Intelligence (Wechsler, 1999). In the vocabulary subtest, the participant simultaneously hears and sees a word and is asked to explain its meaning. In the matrix reasoning subtest, participants are shown a matrix of visual stimuli with one piece missing; they are required to select the missing visual stimulus from five response options.

### Interventions

#### Working Memory Training (Cogmed RM, Pearson)

Twenty participants were randomly allocated to the WM training, which consists of 25 sessions completed 5 days per week for 5 weeks; participants completing at least 20 sessions over 8 weeks are regarded as complying with training. Each 30–45 min session includes eight computerized tasks that require visuo-spatial or verbal WM. Each activity includes 15 trials and the difficulty level (number of items to be remembered) is adjusted trial-by-trial. The program generates an ‘index of improvement’ which provides a measure of the progress made on one verbal and one visuo-spatial task over the training period. Previous work indicates that the mean index of improvement for children aged 7–17 years is 27 (*SD* = 13; Cogmed Coaching Manual).

Thirteen participants (Mean age = 13 years, 0 months; two male) met criteria for training compliance, attending a mean of 22.77 sessions (*SD* = 2.52, Range = 20–25) and achieving a mean index of improvement of 22.62 (*SD* = 14.18, Range = 6–49). Seven participants (Mean age = 13 years, 1 month; two male) did not complete training due to low motivation, attending a mean of 7.00 sessions (*SD* = 4.16, range = 1–15). Completers and non-completers were compared on T1 measures and significant differences between groups were observed for IQ, *t*(18) = 2.95, *p* = 0.009; IQ scores were significantly greater in the completers (*M* = 100.08, *SD* = 5.91, range = 88–108) compared with the non-completers (*M* = 92.29, *SD* = 5.02; Range = 86–99).

#### FRIENDS for Life (Barrett, 2005)

Twenty participants were randomly allocated to the CBT intervention, which consisted of 10 one-hour sessions conducted twice per week for 5 weeks. The intervention followed a manual which incorporates small group activities on feelings, thoughts, relaxation techniques, problem solving and coping strategies. Nineteen participants (Mean age = 12 years, 11 months; six males) complied with training and attended a mean of 9.37 sessions (*SD* = 0.83, range = 8–10); one female participant did not comply with training due to scheduling difficulties and low attendance (*n* = 4 sessions).

### Procedure

All aspects of the study were reviewed and approved by the internal university ethics and research governance procedures and complied with the ethical principles of the British Psychological Society. The screening questionnaires were administered in groups of 20–30 individuals during the school day supervised by teaching staff. Participants recruited into the interventions completed the outcome measures in two sessions during the school day at each time point. After completing T1 measures, participants were matched into pairs based on their RCMAS total anxiety scores. Using a computerized random number generator, one member of each pair was allocated to the WM group and the other member allocated to the CBT group by the second author who was blind to the identity or characteristics of the participants.

The WM training sessions were completed on a school or home computer; a trained Cogmed coach monitored progress following every completed session using the online system provided by Cogmed. The coach met with all participants who complied with training at least twice per week to provide motivation and feedback on progress. The CBT sessions were completed in small groups (4–6 individuals) in a classroom at school, led by a researcher trained to run the FRIENDS for Life program. Monetary reward were provided for both groups with £1 awarded for every session completed and an additional £5 awarded for participants completing all sessions.

### Data Analysis

For each outcome measure (WM, anxiety, attentional control and attention to threat) we analyzed data between groups (WM, CBT) and over Time (T1, T2, T3) using repeated measures ANOVAs, where a group by time interaction would indicate a differential impact of the interventions over time. Exploratory data at T1 showed that IQ was correlated with WM scores (*r* = 0.47, *p* = 0.01 and *r* = 0.37, *p* = 0.037 for near and distant WM tasks respectively); therefore IQ was entered as a covariate in all WM analyses. For all analyses we report effect sizes and 95% confidence intervals around group means. The reported analyses are based on the participants who complied with training (completer analysis). For non-completers (*N* = 8), an intention-to-treat (ITT) analysis was also conducted for each outcome variable using the last-observation-carried-forward method (see Streiner, 2002). Five non-completers provided a full set of outcome measures at T1 only and the scores for these participants were carried forward to T2 and T3. Three non-completers provided outcome measures at all time points and these were used in the ITT analyses. All findings reported below for the completer analysis were replicated in the ITT analysis and these results are therefore not reported further. All statistical tests were two-tailed with an alpha level of 0.05.

## Results

Analyses were carried out to consider group differences in IQ and between IQ with outcome variables. There was no group difference in IQ (*t* < 1 and *p* > 0.05). **Table 1** presents the characteristics of the WM (*n* = 13) and CBT (*n* = 19) group at all three time points; there were no significant differences between the WM and CBT groups on any of the T1 measures in the completer or ITT samples (all *t*s > 1.5; *p*s > 0.1).

**Table 1:** Mean (SD) and range of working memory (WM) (% near and distant correct), anxiety symptoms, stroop interference score (ms), attentional bias score (ms) at measures at time 1 (T1- pre-intervention), time 2 (T2 – post-intervention) and time 3 (T3 – follow-up) in the WM and CBT groups.

	WM group^∗∗^						CBT group^#^					
		
	T1		T2		T3		T1		T2		T3	
						
	*M*	*SD*	*M*	*SD*	*M*	*SD*	*M*	*SD*	*M*	*SD*	*M*	*SD*
**Working memory tasks**								
Near	49.39	7.04	58.69	10.08	58.97	10.53	45.07	9.91	46.97	12.49	48.27	10.98
Distant	59.40	17.37	69.41	14.93	68.37	15.27	59.94	10.16	63.86	13.16	67.54	14.60
**Anxiety measures**								
Trait anxiety	21.92	7.33	17.76	7.15	14.00	7.30	18.00	8.76	13.89	8.78	14.00	9.09
Test anxiety	74.54	15.14	70.77	19.25	61.77	19.00	74.37	19.13	68.79	19.42	70.79	24.11
**Attention measures**								
Interference^∗^	97.55	19.46	57.13	16.86	57.39	14.87	85.96	16.54	66.83	37.51	42.76	12.64

**Both groups (*n* = 32)**												

	**T1**				**T2**				**T3**			

Bias toward^@^	44.61		35.63		6.42		41.03		-24.46		-24.46	
Bias away	-38.53		30.44		15.15		59.23		12.89		12.89	


*^∗∗^*N* = 13 participants; ^#^*N* = 19 participants, ^∗^interference scores indicate the RT (ms) differences between incongruent versus congruent trial (increased positive scores reflect greater interference) and *N* = 18 participants completed the stroop test in the CBT group because one participant reported color blindness. ^@^Indicates bias toward threat and bias away from threat collapsed across groups.*

### Training Effects

#### Working Memory (Near)

**FIGURE 1:**
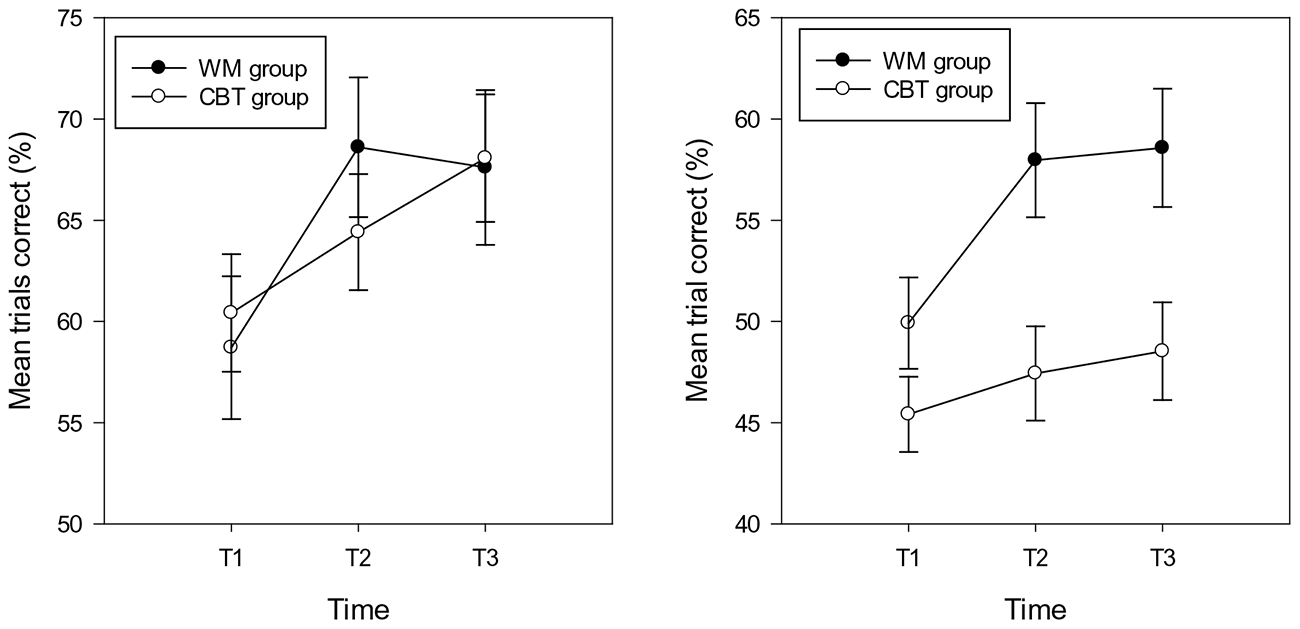
**Percentage of trials correct (and standard errors) in the near (right hand graph) and distant (left hand graph) working memory tasks in the WM and CBT intervention groups at each time point**.

Analyses were carried out separately for the composite (spatial and verbal) WM near and distant scores. The repeated measures ANCOVA (controlling for IQ) on the percentage of trials correct in the near WM tasks showed a significant effect of group [*F*(1,29) = 6.15, *p* = 0.019, $\eta_{p}^{2}$ = 0.18]^3^ . The percentage of trials correct was higher in the WM group (*M* = 55.16, *SD* = 8.73, *CI*: 50.07 – 60.24) compared with the CBT group (*M* = 47.14, *SD* = 10.43, *CI*: 42.94 – 51.34). The main effect of time approached significance [*F*(2,29) = 2.85, *p* = 0.066, $\eta_{p}^{2}$ = 0.09], indicating a lower percentage of trials correct at T1 (*M* = 47.16, *SD* = 8.99, *CI*: 44.17 – 50.15) compared with T2 (*M* = 52.72, *SD* = 12.81, *CI*: 48.98 – 56.46) and T3 (*M* = 53.56, *SD* = 11.89, *CI*: 49.70 – 57.42) (T2 T3 ns.). The interaction between time and group was significant [*F*(1,29) = 5.82, *p* = 0.005, $\eta_{p}^{2}$ = 0.17], highlighting group differences at T2 (*M* = 57.96, *SD* = 10.08, *CI*:52.19 – 63.75 and *M* = 47.47, *SD* = 12.50, *CI*: 42.69 – 52.25 for the WM and CBT groups respectively) and T3 (*M* = 58.58, *SD* = 10.53, *CI*: 52.61 – 64.55 and *M* = 48.54, *SD* = 10.98, *CI*: 43.60 – 53.48 for the WM and CBT groups) respectively. The group difference at T1 (respective means: *M* = 48.92, *SD* = 7.04, *CI*: 44.29 – 53.54 and *M* = 45.40, *SD* = 9.92, *CI*: 41.58 – 49.23 was not significant; see **Figure 1**). Within group analyses for time were not significant for the WM group (*F* < 2.5, *p* > 0.1) or the CBT group (*F* < 1, *p* > 0.1).

#### Working Memory (Distant)

**FIGURE 2:**
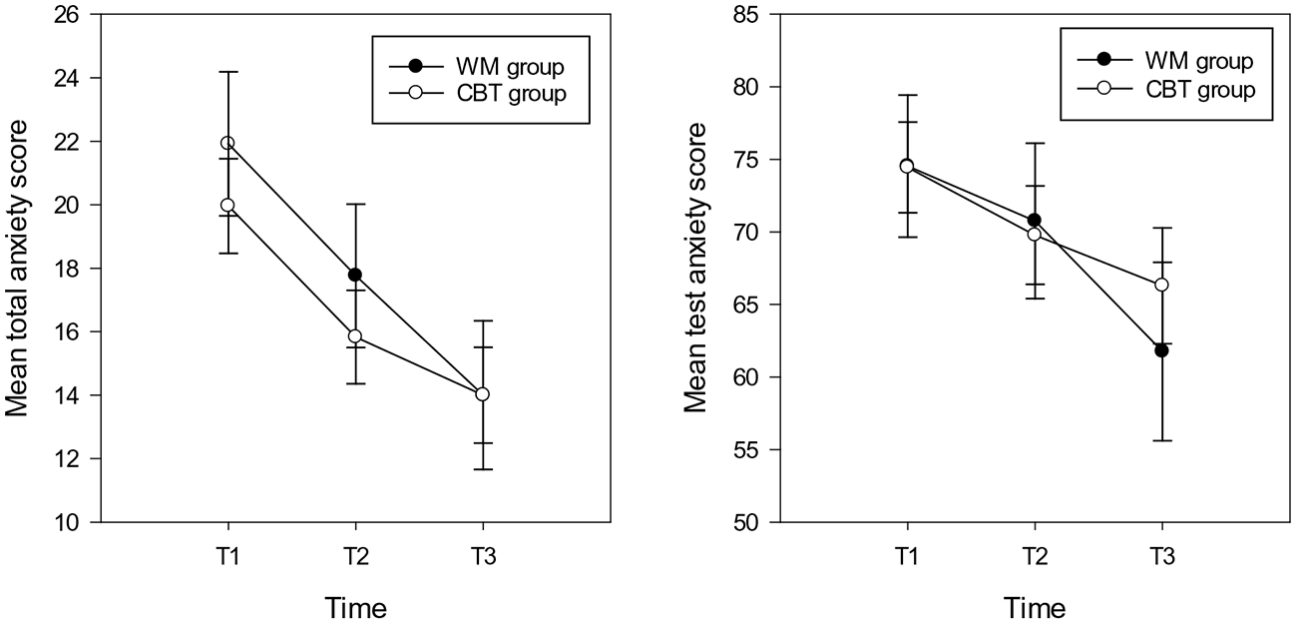
**Mean total anxiety scores (and standard errors) for total anxiety (left hand graph) and test anxiety (right hand graph) in the WM and CBT intervention groups at each time point**.

The repeated measures ANCOVA (controlling for IQ) on the percentage of trials correct in the distant WM tasks showed no main effect of group or time (*F*s < 1, *p* > 0.1). In addition, the interaction between time and group was not significant (*F* < 1.5, *p* > 0.1); see **Figure 2.** The mean accuracy scores for the WM and CBT groups at T1, T2, and T3 were: 58.70, 68.61, 67.61, and 6.42, 64.41, 68.07; see **Figure 1.**

#### Anxiety Symptoms

For total anxiety symptoms the repeated measures ANOVA showed a main effect of time [*F*(2,30) = 16.71, *p* < 0.001, $\eta_{p}^{2}$ = 0.36], the mean anxiety scores at T2 (*M* = 15.47, *SD* = 8.26, *CI*: 12.83 – 18.84) and T3 (*M* = 14.00, *SD* = 8.30, *CI*:10.90 – 17.10) were significantly lower compared with T1 (*M* = 19.96, *SD* = 31, CI: 16.94–22.98) (T2 T3 ns following Bonferroni correction). There was no main effect of group and no interaction between group and time (*F*s < 2.5, *p* > 0.1; see **Figure 2**). With respect to test anxiety, the analysis showed a main effect of time [*F*(2,30) = 4.73, *p* = 0.012, $\eta_{p}^{2}$ = 0.14], highlighting more reported symptoms of test anxiety at T1 (*M* = 74.43, *SD* = 17.35, *CI*: 67.97 – 80.93) compared with T3 (*M* = 66.28, *SD* = 22.30, *CI*: 58.12 – 74.44). There was no difference between T1 and T2 (*M* = 69.78, *SD* = 19.06, *CI*: 62.67 – 76.89) or T2 and T3 test anxiety scores. There was no main effect of group (*F* < 1, *p* > 0.1). The interaction between group and time approached significance [*F*(2,30) = 2.45, *p* = 0.095, $\eta_{p}^{2}$ = 0.08]; indicating that there were no significant differences between any time point for the CBT group. However, for the WM group, time differences were evident between T3 with both other time points (T1 T2 ns; see **Figure 2**).

#### Inhibitory Control

##### Task performance

A one way (stimulus type: congruent, incongruent, neutral) repeated measures ANOVA on RTs in the Stroop task at T1 revealed a typical congruency effect. There was a main effect of stimulus type [*F*(1.36,40.81) = 36.60, *p* < 0.001, $\eta_{p}^{2}$ = 0.55], where RTs were significantly longer in the incongruent condition (*M* = 806.12 ms, *SD* = 133.13, *CI* = 757 – 854) compared with the congruent (*M* = 715.29 ms, *SD* = 106.42, *CI* = 676 – 754, *p* < 0.001) and neutral conditions (*M* = 741.87 ms, *SD* = 111.27, *CI* = 701 – 782, *p* < 0.001). RTs in the congruent conditions were also significantly faster than the RTs in the neutral condition (*p* < 0.001).

**FIGURE 3:**
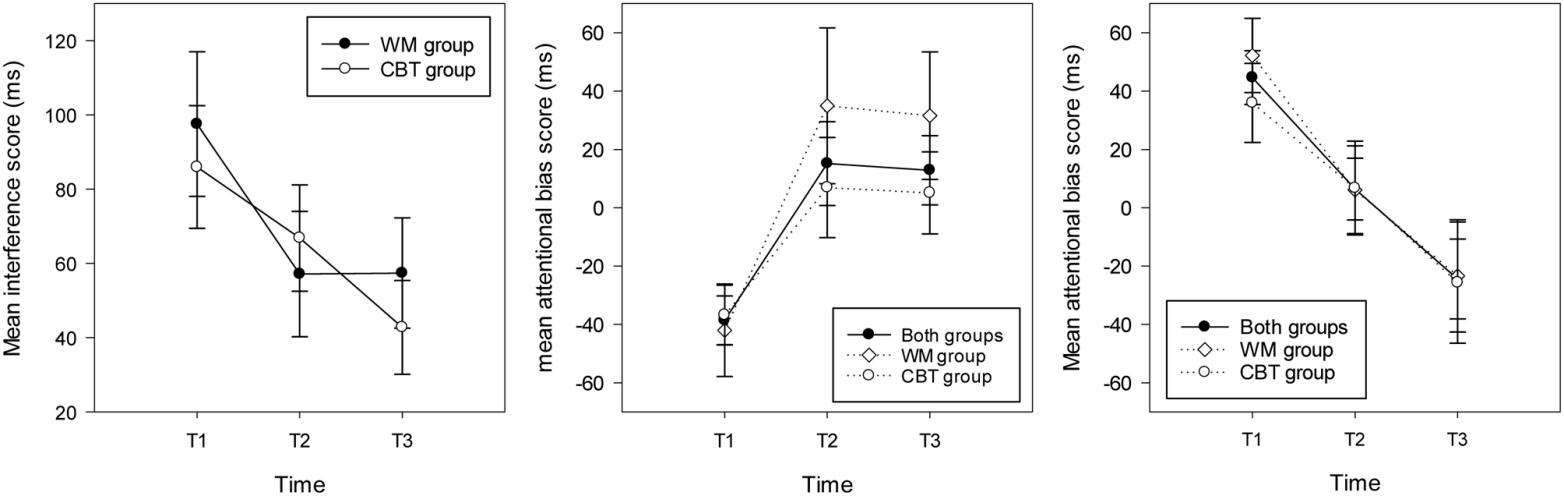
**Mean interference scores (and standard errors) in the stroop task (left hand graph) and attentional bias scores (and standard errors) in the WM group, the CBT and both groups combined for individuals attending toward threat (right hand graph) and away from threat (middle graph) at each time point**.

The repeated measures ANOVA on interference scores revealed no significant main effect of group and no interaction between group and time (*F*s < 1, all *p*s > 0.1). There was a main effect of time [*F*(2,60) = 51, *p* = 0.003, $\eta_{p}^{2}$ = 0.18] showing significantly higher interference scores at T1 (*M* = 90.82 ms, *SD* = 69.25, *CI* = 65 – 116) compared with T2 (*M* = 62.76, *SD* = 59.99, *CI* = 41 – 85) and T3 (*M* = 48.90 ms, *SD* = 53.23, *CI* = 29 – 68); see **Figure 3.**

#### Threat Bias

##### Task performance

A one way (probe position: congruent, incongruent, neutral) repeated measures ANOVA was conducted on RTs in the dot probe paradigm at T1 in order to understand baseline task performance. The results revealed no significant effect of probe position on RTs (*F* < 1, *p* > 0.1; congruent: *M* = 715.71 ms, *SD* = 163.63; incongruent: *M* = 716.25 ms, *SD* = 163.42; neutral: *M* = 722.15 ms, *SD* = 162.57).

Considering group differences and attentional bias to threat, the results showed no effect of group [*F*(1,30) = 2.14, *p* = 0.12, $\eta_{p}^{2}$ = 0.02]. In addition, there was no main effect of time and the interaction between time and group was not significant (in both cases *F* < 1 and *p* > 0.1). The respective mean bias scores (and SD) for threat for the WM and CBT intervention groups at each time point was 15.97, (*SD* = 64.90, *CI* = -14.43 – 46.37), 17.23 (*SD* = 51.94, *CI* = -11.93 – 46.38), -2.26 (*SD* = 59.75, *CI* = -33.03 – 28.51) and -10.01 (*SD* = 44.65, *CI* = -35.16 – 15.13), 6.85 (*SD* = 51.17, *CI* = -17.27 – 30.97), -6.24 (*SD* = 50.39, *CI* = -31.70 – 19.21). Further exploration of the T1 attentional bias scores showed that across the two intervention groups there were two types of participant at baseline; those that attended toward threat (bias score > 0, *n* = 15) and those that attended away from threat (bias score < 0, *n* = 17). Therefore, analyses were run separately for the two types of threat bias. Because sample sizes were small analyses were collapsed across groups to explore the effect of time on bias scores.^4^

For the participants who attended to threat at T1, the ANOVA revealed a main effect of time [*F*(2,28) = 8.26, *p* = 0.002, $\eta_{p}^{2}$ = 0.37], highlighting significantly higher bias scores at T1 (*M* = 44.63 ms, *SD* = 35.64, *CI*: 24.89 – 64.36) compared with T2 (*M* = 6.42, *SD* = 41.03, *p* = 0.082, *CI*:-16.29 – 29.14) and *T3* (*M* = -24.46 ms, *SD* = 52.96, *p* = 0.008, *CI*: -53.78 – 4.68). Considering each time point separately, one sample *t*-tests showed a significant bias toward threat at T1 [*t*(14) = 4.85, *p* < 0.001], no bias at T2 (*t* < 1, *p* > 0.1) and a marginal trend for a bias away from threat at T3 [*t*(14) = 1.79, *p* = 0.095; see **Figure 3**]. For participants who attended away from threat at T1, the repeated measures ANOVA revealed a main effect of time [*F*(2,32) = 6.75, *p* = 0.004, $\eta_{p}^{2}$ = 0.30] with significantly higher (avoidant) bias scores at T1 (*M* = -38.36 ms, *SD* = 34.44, *CI*: -56.07 – -20.65) compared with T2 (*M* = 15.16, *SD* = 59.24, *CI:*-15.29 – 45.61) and T3 (*M* = 12.88 ms, *SD* = 48.93, *CI:*-12.28 – 38.03). One sample *t*-tests indicated that there was a significant bias away from threat for this group of participants at T1 [*t*(16) = 4.59, *p* < 0.001], and no bias at T2 or T3 (*t*s < 1.5, *p* > 0.1); see **Figure 3.**

## Discussion

The current study provides preliminary evidence to demonstrate reductions in self-report anxiety symptoms, anxiety-related cognitive biases for threat and increased inhibitory control following WM and CBT interventions for adolescents who reported elevated anxious affect and low attentional control. In addition, the WM group showed better performance post intervention on tasks similar to those that were trained, compared to the CBT group. The findings link to previous intervention studies which have found that adults with elevated depression symptoms benefitted from a WM intervention to show improved performance on WM tasks (Owens et al., 2013). In addition, they are consistent with research which has shown that young people with social and emotional behavioral difficulties who showed increased performance on WM tasks, better inhibitory control and fewer symptoms of test anxiety after completing a WM training intervention compared with a passive control group (Roughan and Hadwin, 2011). The current study extends previous research to show reduced attentional biases to threat following CBT and WM interventions. The findings fit with a broader literature highlighting the effectiveness of a WM training intervention on the reduction of symptoms associated with developmental psychopathology including inattention, hyperactivity and oppositional behavior (e.g., Klingberg et al., 2005; Beck et al., 2010).

Consistent with previous studies, the findings reported here showed reduced anxiety symptoms following a CBT intervention (e.g., Stallard et al., 2008). A recent meta-analysis highlighted that CBT is an effective treatment for anxiety reduction in children and adolescents compared to wait-list control groups (James et al., 2015). These authors noted, however, that few studies have compared CBT to active control groups receiving other forms of psychological intervention. The current study showed that the magnitude of the reduction in anxiety symptoms did not differ between the WM training and CBT intervention groups. They provide tentative evidence to suggest that attentional training could be a viable alternative or complementary intervention for children and adolescents with elevated anxious affect. Moreover, they fit with current studies suggesting that interventions that focus on attentional processes in anxiety might provide an important supplement to CBT and where this alternative approach could be most effective for individuals who are not responsive to more traditional treatment approaches (e.g., Bechor et al., 2014; review by Lowther and Newman, 2014).

The current study extends previous research to demonstrate that WM training and CBT led to increased inhibitory control post-intervention. In previous studies, wait-list control groups have shown relative stability in inhibitory control over a 3 months period (Roughan and Hadwin, 2011). Past research has not typically considered the impact of CBT on measures of attentional control. The current findings link to one study with adults with a clinical diagnosis of obsessive–compulsive disorder and who completed a CBT intervention. This group of adults showed cognitive deficits in set shifting, non-verbal memory and flexible behavior prior to treatment and these difficulties were no longer evident following CBT (Kuelz et al., 2006). In addition to improved inhibitory control, the current study found that across both groups adolescents showed reductions in attentional biases (characterized as biases toward or away from threat stimuli), indicating increased attention on task goals post intervention. This finding extends the previous literature (e.g., Waters et al., 2012) to indicate that reductions in attentional bias were not restricted to individuals completing CBT, but were also evident following a WM intervention.

Despite the growing emphasis on attentional control deficits in cognitive models of anxiety (e.g., Eysenck et al., 2007) and related research (Susa et al., 2012), the focus of recent interventions has been on modifying attentional biases to threat via ABM (e.g., Pérez-Edgar et al., 2014), rather than on improving attentional control more broadly. The results in the current study represent an important first step in the development of attention based interventions for adolescents who experience elevated anxiety. They suggest that biases for threat can be impacted via improved attentional control and in the absence of moderating attentional threat biases directly. Because improved attentional control and a reduction in threat biases was evident in both intervention groups, the results suggest that positive changes in anxiety symptoms can result from improved attentional control, as well as following more traditional CBT. Research with larger sample sizes would allow further examination of pathways to change via different interventions.

The proposition that increased attentional control (via WM training) can impact positively on anxiety symptoms is consistent with a broader literature that has highlighted the role of the PFC in emotional regulation (e.g., Davidson et al., 2000). It also links to related studies that have found a negative association between PFC activation with brain regions linked to fearful responding, including the amygdala (Etkin et al., 2006; Hare et al., 2008). For example, research has demonstrated that adults who report elevated anxiety show reduced ability to utilize attentional processes associated with the PFC (including the Anterior Cingulate Cortex and the lateral PFC) and where this pattern of activation is argued to maintain threat biases in anxiety (Öhman, 2005; reviews by Bishop, 2007).

Previous research also suggests that the reduction in anxiety symptoms following CBT has been associated with increased activation of the PFC and associated improvements in emotional regulation when completing cognitive tasks. One goal of CBT is positive re-framing (Cowart and Ollendick, 2010). The reduction in attention biases in the current study following CBT links to studies which have found that positive re-appraisal of negative stimuli is associated with increased activation in the PFC and reduced activation in the amygdala. For example, Ochsner et al. (2002) asked participants to attenuate emotional responses to negative picture stimuli (versus inspect them as they typically would). They showed that brain activation in the Dorsolateral PFC was associated with stimulus reappraisal and where this process was inversely linked to amygdala activation (Ochsner et al., 2002; see also Banks et al., 2007). Consistent with this finding, Maslowsky et al. (2010) showed that following a CBT intervention young adolescents with a primary diagnosis of generalized anxiety disorder showed increased activation in the right ventrolateral PFC and the authors argued that this activation reflected top–down regulation of negative emotion following treatment. To understand mechanisms of change, future research using WM and CBT interventions would benefit further from exploring neurocognitive change following treatment (e.g., Owens et al., 2013).

The current study had a number of further strengths. The inclusion of a 3-months follow-up assessment was important in highlighting that improvements on all outcome measures were maintained over an extended period of time. A further notable finding of the current study was to identify baseline characteristics associated with drop-out from the WM intervention. The results indicated that completion of the WM intervention was particularly challenging for individuals with lower IQ, raising the possibility that a reduction in the intensity of the intervention (i.e., the frequency and duration of sessions) could be beneficial for some young people. While this finding was important, the challenges associated with the WM group led to the attrition rate being higher than the CBT group. Although there were no differences on baseline measures between the individuals completing each intervention, we cannot rule out the possibility that the WM group represented a particularly motivated group who were able to overcome the challenges of the WM training (see Jaeggi and Buschkuehl, 2014). A further limitation in the current study was the absence of a wait-list control group. Previous research has consistently shown benefits of WM and CBT training versus passive control groups (James et al., 2015; Spencer-Smith and Klingberg, 2015) and researchers have argued that their inclusion can exaggerate treatment effects (Cunningham et al., 2013). However, the inclusion of a passive control group in the context of scoping trials does have some benefit in understanding the stability of outcome attentional measures over time and in the absence of an intervention. These limitations highlight the need for larger scale studies with increased sample sizes to account for high attrition rates and that include WM, active control and passive control groups.

One further difficulty in the current study was the lack of generalization of WM training to untrained WM tasks. A recent meta-analysis concluded that there was evidence of reliable near transfer effects on measures of verbal and visuospatial WM in the short term after training; however, there was no evidence of distant transfer effects on measures of cognitive ability or educational achievement (Melby-Lervåg and Hulme, 2013). It is possible that the current distant WM tasks utilized in the current study were not sensitive to differences between interventions, therefore, future research should aim to ensure that a range of untrained tasks is included in the evaluation of WM interventions. In addition, the current study focused on trait measurements of anxiety. And Supplementary Analysis revealed a mixed profile on outcomes linked to additional measures of negative affect and educational achievement. Though preliminary, they showed no effect of either intervention on self-report state anxiety, though some positive change in symptoms of depression and achievement scores in both groups. Consistent with theoretical accounts of anxiety, a recent review argued that state anxiety can moderate the impact of trait anxiety on attentional tasks, making it an important index of treatment outcome (Robinson et al., 2013). Moreover, it highlighted the complex association between anxiety and performance on WM tasks and where better performance can reflect increased individual effort and/or task cognitive load (see also Eysenck and Derakshan, 2011). Future research should aim to capture potential performance moderators using objective measures of effort and emotional regulation.

## Conclusion

The current study outlines preliminary data which indicates that both WM and CBT interventions were effective in reducing anxiety symptoms in young people. While the study reflects a small sample size, its findings support the notion of a “proof of concept” in training WM (Gathercole et al., 2012) that indicate a broader positive impact on increased inhibitory control and attentional biases for threat. The novel findings should encourage the use of larger scale replication RCTs in clinical and educational settings that place greater emphasis on understanding the key mechanisms of change, as well the impact of baseline characteristics on attrition and treatment outcomes (Jaeggi and Buschkuehl, 2014).

## Author Contributions

Both authors made substantial, direct and intellectual contribution to the work, and approved it for publication.

## Conflict of Interest Statement

The authors declare that the research was conducted in the absence of any commercial or financial relationships that could be construed as a potential conflict of interest.

## Footnotes

1 Note that a comment posted in the same journal and following the publication and re-analysis of data in this article argued that there was little evidence of WM training on positive change in daily life (Dovis et al., 2015).

2 Participants also completed outcome measures for academic achievement, depression, and state and anxiety. The results associated with these measures were not a focus of the current paper and are therefore reported in Supplementary Analyses.

3 A *post hoc* power calculation for two independent samples and for a sample size of 13 for near WM tasks (related to mean T1T2 change in the WM group (mean = 9.29) versus the CBT group (mean = 1.90) indicated a power of 0.91.

4 Note that the addition of the group in both analyses did not change the main effect of time and in both cases the main effect of group and the interaction between group and time was not significant (all *F*s < 1 and all *p*s > 0.1).
