# Supplementary material for: Working Memory Training and CBT Reduces Anxiety Symptoms and Attentional Biases to Threat: A Preliminary Study
Source: Front Psychol. 2016 Feb 2;7:47. doi: 10.3389/fpsyg.2016.00047 (PMC4735443; doi:10.3389/fpsyg.2016.00047)
Supplement: Supplementary file 1 [file Data_Sheet_1.DOCX]

Appendix A

*Flow diagram of the progress through the phases of the randomised trial*

Total number of pupils identified for screening: n = 1560

1. Assessed for eligibility via screening: n = 640
2. Declined to participate in screening: n = 920

Excluded (n = 600):

1. Not meeting screening criteria (n = 480)
2. Not meeting exclusion criteria (n = 14)
3. Declined to participate in intervention (n= 106)

Randomised: (n = 40)

Allocated to WM intervention (n = 20)

1. Received allocated intervention (n = 13)
2. Did not receive allocated intervention (n = 7 due to scheduling issues or lack of motivation)

Allocated to CBT intervention (n = 20)

1. Received allocated intervention (n = 19)
2. Did not receive allocated intervention (n = 1 due to scheduling issues)

Lost to post-test (n= 1 due to discontinuation of intervention)

Lost to follow-up (n= 1 due to discontinuation of intervention)

Lost to post-test (n= 4 due to discontinuation of intervention)

Lost to follow-up (n = 4 due to discontinuation of intervention)

Data available for analysis:

13 completers

3 non-completers (data available from all three time points)

4 non-completers (last observation carried forward from T1)

Data available for analysis:

19 completers

1 non-completer (last observation carried forward from T1)
