# Supplementary material for: Working Memory Training and CBT Reduces Anxiety Symptoms and Attentional Biases to Threat: A Preliminary Study
Source: Front Psychol. 2016 Feb 2;7:47. doi: 10.3389/fpsyg.2016.00047 (PMC4735443; doi:10.3389/fpsyg.2016.00047)
Supplement: Supplementary file 2 [file Image_1.PDF]

Supplementary analyses

We explored the effect of intervention on self-report symptoms of depression, state anxiety and performance in school tests.

Method

Measures

We measured symptoms of depression using the subscale in the Revised Children's Manifest Anxiety Scale (Chorpita, Yim, Moffitt, Umemoto, & Francis, 2000). Here children are asked to respond to "never", "sometimes" "often", "always" to 10 items (e.g., "It feels like nothing is much fun anymore") and with a possible score range from 0-30. We measured current (state) anxiety using the State-Trait Anxiety Inventory for Children (Spielberger, Edwards, Lushene, Montuori, & Platzek, 1973). This scale includes 20-item scales where participant are asked to respond to each item (e.g., "I feel calm", "I feel nervous") on a 4 point scale ("not at all", "somewhat" "moderately so" and "very much so"); generating a possible score range from 20 to 80. Academic ability was measured using two subscales (maths and spelling) of the Wide Range Achievement test (WRAT-4; Wilkinson & Robertson, 2006) were used. In the spelling test, participants hear a series of increasingly complex words that they have to spell (write down). In the maths test they are given 15 minutes to complete as many maths problems as they can.

Results

The result showed no effect of time for either intervention group with state anxiety following experimental tasks at each time point and the interaction between time and group was also not significant (in all cases $F < 2$ and $p > .1$). Considering self-report depression symptoms, the results showed a main effect of time for symptoms of depression ($F(2,30)=4.20$, $p = .020$, $\eta^2 = .12$) highlighting that symptoms of depression were significantly higher at T1 (mean = 9.51) compared with T3 (mean = 7.43). (T1 T2 (8.07) and

T2 T3 ns). The main effect of group and the interaction between group and time for depression symptoms was not significant (in both cases $F < 1$ and $p > .1$). See Figure SA1 below for state anxiety, depression scores for each group and at each time point .

Considering change over time and between groups in maths and spelling. The results for spelling showed a main effect of time ($F(2,30)=13.05$, $p < .001$, $\eta 2 = .30$), highlighting differences in spelling between T1 (mean = 36.56) and T2 (mean = 37.06) with T3 (38.68; T1T2 ns). The main effect of group and the interaction between group and time was not significant (Fs < 2 and ps > .1). For maths, there was also a main effect of time F (2, 30) =4.59, $\eta 2 = .16$), showing a difference between T1 (mean = 35.15) and T2 (mean = 37.06) (T1T3 (mean = 35.87) and T2T3 ns). The main effect of group was not significant ($F < .1$, $p > .1$). And the interaction between time and group approached significance (F (2, 30) = 2.30, $p = .091$, $\eta 2 = .08$), highlighting marginal differences between T2 (mean = 37.38) with T1 (mean = 35.15) and T3 (mean = 34.69; T1T3 ns) for the WM intervention only; see Figure SA2).

Figure SA1

Self-report symptoms of depression (left hand graph) and state anxiety (right hand graph) for the WM and the CBT intervention group at each time point

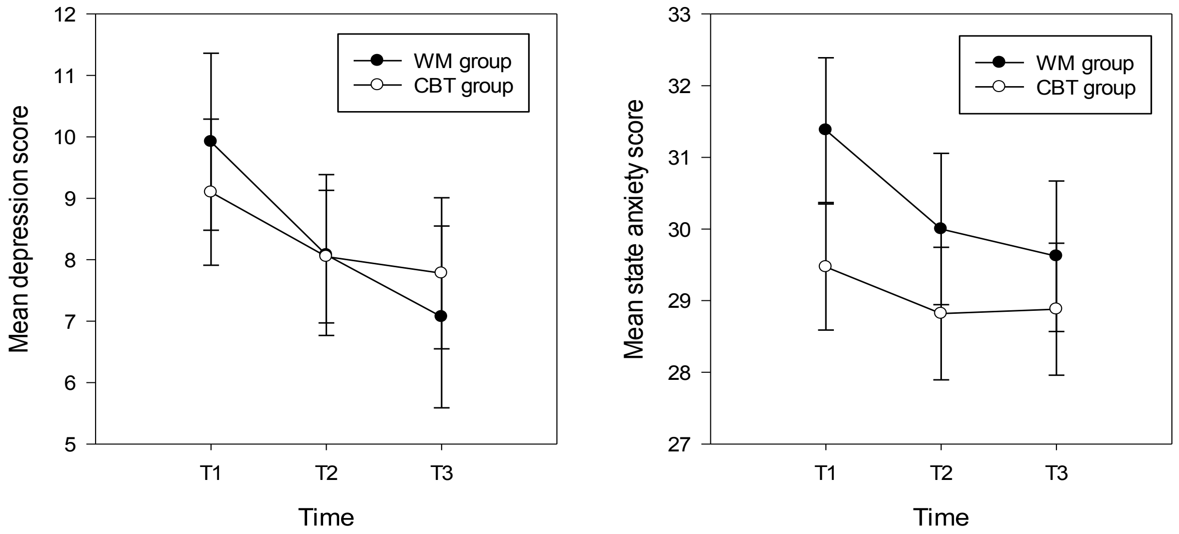

Figure SA2

Measures of spelling and maths for the WM and the CBT intervention group at each time point

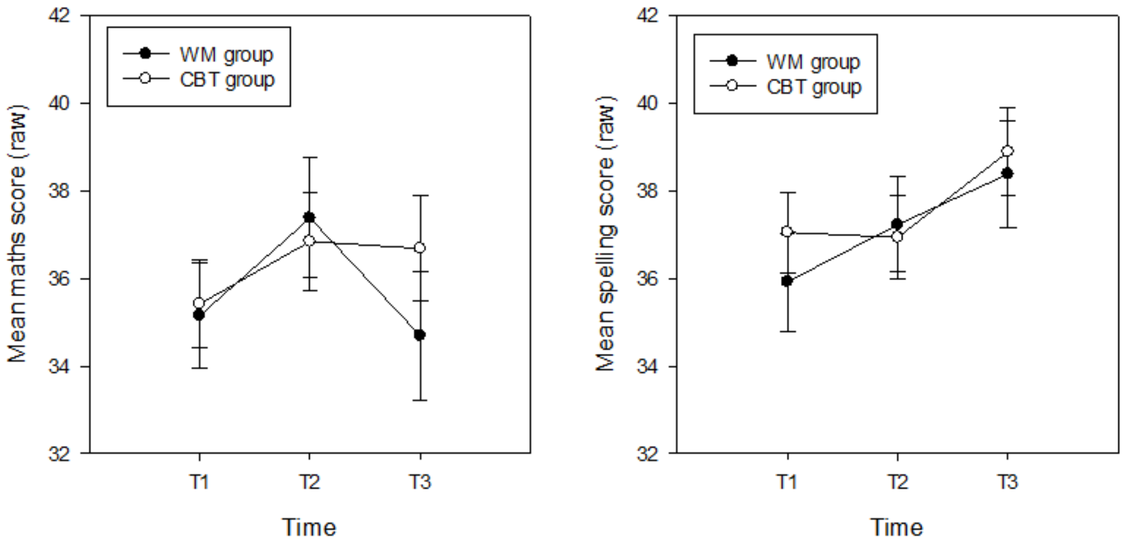

References

Chorpita, B.F., Yim, L., Moffitt, C., Umemoto, L.A. & Francis, S.E. (2000). Assessment of symptoms of DSM-IV anxiety and depression in children: A revised child anxiety and depression scale. *Behaviour Research and Therapy, 38*, 835-855. DOI: 10.1016/S0005-7967(99)00130-8.

Spielberger, C., Edwards, C.D., Lushene, R., Montuori, J. & Platzek, D. (1973). *State-trait Anxiety Inventory for Children*. Mental-Health Systems Inc.

Wilkinson, G.S. & Robertson, G.J. (2006) *Wide Range Achievement Test* (Fourth Edition). Pearson Ltd.
